# Effects of Dietary Vitamin E, Vitamin C, Selenium and Their Combination on Carcass Characteristics, Oxidative Stability and Breast Meat Quality of Broiler Chickens Exposed to Cyclic Heat Stress

**DOI:** 10.3390/ani12141789

**Published:** 2022-07-12

**Authors:** Manca Pečjak, Jakob Leskovec, Alenka Levart, Janez Salobir, Vida Rezar

**Affiliations:** Department of Animal Science, Biotechnical Faculty, University of Ljubljana, 1000 Ljubljana, Slovenia; manca.pecjak@bf.uni-lj.si (M.P.); jakob.leskovec@bf.uni-lj.si (J.L.); alenka.levart@bf.uni-lj.si (A.L.); janez.salobir@bf.uni-lj.si (J.S.)

**Keywords:** vitamin E, vitamin C, selenium, broiler, cyclic heat stress, meat quality, meat oxidative stability

## Abstract

**Simple Summary:**

High ambient temperatures present challenging environmental factors in the poultry meat industry, causing heat stress. Heat stress impairs animal health and welfare, growth performance, and productivity, and deteriorates meat quality and its oxidative stability, resulting in economic losses. To mitigate the negative effects of heat stress, several nutritional strategies have been proposed, with vitamin and mineral supplementation being one of the most promising. Several studies reported that the addition of vitamins E and C, as well as selenium, to broiler diets improved the production performance and meat quality of broilers reared under heat stress. Due to the synergistic effects of these antioxidants against the oxidative damage to lipids, combined supplementation could be even more effective in alleviating the symptoms of heat stress on meat quality than supplementation alone, but this has not yet been studied. The results of the present study indicate positive effects of the supplementation with vitamin E on the oxidative stability of breast meat. However, no synergistic effects of the added antioxidants on the measured parameters were observed.

**Abstract:**

The present study was conducted to investigate the effects of dietary supplementation with vitamin E, vitamin C, and Se, alone or in combination, on carcass characteristics, oxidative stability and meat quality parameters of breast meat from broilers exposed to cyclic heat stress (HS), and stored under different conditions. A total of 120 one-day-old male Ross 308 broilers were randomly assigned to six dietary treatments: NRC (minimal nutrition requirements), AVI (commercial nutritional recommendations for Ross 308 broilers), AVI + vitE (as AVI + 200 IU vitamin E/kg feed), AVI + vitC (as AVI + 250 mg vitamin C/kg feed), AVI + Se (as AVI + 0.2 mg Se/kg feed), and AVI + ECSe (as AVI + vitE + vitC + Se). From day 26 onwards, all birds were exposed to a high ambient temperature (31 °C) for 8 h per day. The results showed that dietary vitamin E alone or in combination with vitamin C and Se significantly increased the α-tocopherol content and decreased the malondialdehyde (MDA) content in breast meat. Although no obvious synergistic effects of the added antioxidants were observed, the addition of higher levels of vitamin E, vitamin C and Se to broiler diets had no adverse effects on carcass traits, oxidative stability and meat quality parameters when supplemented either alone or in combination.

## 1. Introduction

In the past decades, we have been facing climate changes associated with the global warming that have significant impacts on the livestock sector, especially poultry production, which is known to be particularly susceptible to environmental stressors [1]. In addition, intensive genetic selection of broiler chickens for a greater growth rate, feed conversion efficiency and larger breast muscle, have resulted in an insufficient ability of birds to control their body temperature in response to fluctuations in the ambient temperature and a high metabolic heat production [2]. One of the most challenging environmental factors causing major economic losses in the poultry industry is high ambient temperatures leading to HS [3]. Heat stress impairs the chickens’ performance and results in higher mortality, lower body weight gain (BWG), better feed conversion ratio (FCR), lower feed intake (FI), and reduced nutrient digestibility [4,5]. High ambient temperatures have also been reported to impair broiler meat quality traits by accelerating the postmortem glycolytic metabolism, resulting in lower muscle pH. This is associated with the occurrence of undesirable changes in broiler breast meat color, particularly lighter meat color, increased drip loss, and decreased water holding capacity (WHC), leading to the occurrence of pale, soft and exudative (PSE) meat [6,7]. Moreover, heat stress increased the lipid oxidation, leading to changes in the muscle fibers structure and increased the deposition of intramuscular fat, which affects consumer acceptance due to decreased tenderness and juiciness of the meat [8].

To mitigate the negative effects of HS in broiler production, various environmental and nutritional strategies have been proposed [1]. When environmental strategies alone are not sufficient, nutritional strategies are required, where the supplementation with various vitamins, minerals, and electrolytes shows the most promising results. Vitamin E is the major lipid-soluble antioxidant that protects cell membranes and tissues from the oxidative damage caused by free radicals [9], especially in the presence of an increased free radical production, HS [10] or dietary stress [11]. Dietary supplementation with vitamin E has been reported to increase its content in muscles, thereby improving the oxidative stability of poultry meat during storage [12] and reducing lipid peroxidation of polyunsaturated fatty acids (PUFA) in membranes, as well as improving the color stability and quality of poultry meat [8]. According to the minimal nutrient requirements for poultry provided by the National Research Council (NRC) [13], the vitamin E requirement for broilers is 10 IU/kg feed, while the current commercial dietary recommendations of Aviagen [14] are in the range of 55 to 80 IU vitamin E/kg feed. In the present study, a higher supplementation of vitamin E was used considering the Aviagen recommendations. This agrees with previous studies in which an average supplementation of 200 mg/kg vitamin E had a positive effect on oxidative status, performance parameters, and meat quality of broilers under HS [10,15].

Another important antioxidant used to alleviate the negative effects of HS is vitamin C. Since vitamin C is not an essential nutrient for poultry, no requirement was established by the NRC [13]. On the other hand, Aviagen [14] has recommended increasing the vitamin supplementation by 2.5% per additional degree Celsius when the ambient temperature exceeds 28 °C. It has been reported that environmental stressors, especially HS, can lead to insufficient endogenous synthesis, increasing the need for exogenous vitamin C [16]. In addition, it has been reported that an average supplementation of 250 mg/kg of vitamin C has positive effects on the performance parameters, carcass characteristics, and meat quality of broilers exposed to HS [17,18]. Vitamin C also plays an important role in restoring vitamin E activity.

In addition to antioxidant vitamins, Se has an important role in the antioxidant defense system as a cofactor for enzymes such as glutathione peroxidase, superoxide dismutase, and thioredoxin reductase. Selenium-dependent glutathione peroxidase detoxifies lipid peroxides and removes free radicals before they damage lipids and proteins in cell membranes, and thus protects them from oxidation damage [19]. Moreover, Se deficiency impairs the absorption and status of vitamin E, which can be prevented by the addition of Se in poultry feed [20]. According to the available studies, selenium supplementation positively affected the antioxidants activity in plasma and tissue, improved growth performance, increased Se content in liver, breast and thigh muscle, decreased lipid oxidation in skeletal muscle and improved meat quality and chemical composition of meat [21,22]. In addition, the combination of Se with vitamin E has been shown to have synergistic effects in reducing lipid oxidation in breast meat of broilers under HS [5]. The commercially recommended Se content in broiler diets to maintain the optimal growth of broilers under normal conditions is 0.3 mg/kg [14], while the minimum requirement determined by the NRC [13] is 0.15 mg/kg. Furthermore, there are no recommendations for additional dietary supplementation in case of exposure to HS. In our study, the Se content in the diet was adjusted to the maximum level allowed in the EU (0.5 mg/kg) [23], although higher Se levels (from 1 to 3 mg/kg) have been used in some studies [5,24] to improve the oxidative stability of broiler breast meat.

To our knowledge, no study has investigated whether simultaneous supplementation with vitamins E, C, and Se has a synergistic effect in mitigating HS and improving the oxidative stability of poultry meat during processing and storage. In contrast, the effects of combined dietary supplementation with the above antioxidants in broiler diets were investigated in our previous research [25], but only in relation to oxidative stress induced by a high PUFA intake. In modern broiler production, both NRC [13] and commercial Aviagen [14] nutritional specifications are used. Furthermore, there is little information on whether NRC [13] requirements are sufficient to mitigate the negative effects of HS compared to Aviagen [14] recommendations.

To test the effectiveness of antioxidants observed under the conditions of HS on carcass characteristics, oxidative stability, and meat quality of broilers, three working hypotheses were formulated: (i) NRC antioxidant levels are less effective in antioxidative protection compared to the Aviagen recommendations; (ii) the supranutritional supplementation above Aviagen recommendation with one of the vitamins E, C and Se has a stronger antioxidative effect than the basal Aviagen recommendation; and (iii) vitamins E, C, and Se act synergistically and provide a better antioxidative protection when supplemented together.

The objective of the present study was to determine whether the supranutritional supplementation with vitamins E, C, and Se improves carcass characteristics, oxidative stability, and meat quality of fresh, chilled, and frozen breast meat of broilers exposed to cyclic HS and whether their combination is even more effective because of the synergistic effects.

## 2. Materials and Methods

All procedures used in the trial were performed according to the scientific and ethical international guidelines for the use and protection of animals in scientific procedures and approved by the Animal Ethics Committee of the Veterinary Administration of the Republic of Slovenia (U34401-9/2019/2).

### 2.1. Broiler Chickens, Husbandry and Temperature Treatments

The present trial was performed at the research facility of the Department of Animal Science, Biotechnical Faculty, University of Ljubljana, Slovenia. One hundred and twenty male Ross 308 broiler chickens were reared from 1 to 43 day of age and randomly divided into 6 experimental groups (according to dietary treatment) on the day of housing. In the completely randomized block design, the experimental groups were replicated twice, resulting in a total of 12 pens with 10 broilers per pen. Each deep litter pen with sawdust (95 × 126 cm) was equipped with a feeder and nipple watering system (5 nipple drinkers/unit). Feed and fresh water were provided *ad libitum* throughout the trial. Chickens were reared under a standard lighting program (18 h light:6 h dark). By day 26, the housing temperature was regulated according to the recommendations for Ross 308 broiler chickens [26], with the ambient temperature maintained at 32 °C during the first week of housing and gradually reduced to 23 °C during the following weeks. On day 26, the ambient temperature varied between 23.5 and 31 °C to trigger conditions of HS. Therefore, broilers were exposed to a temperature of 23.5 °C for 11 h, followed by a temperature increase from 23.5 to 31 °C for 3 h (warm-up phase), a temperature of 31 °C for 7 h (HS phase), and a temperature of 31 to 23.5 °C for 3 h (cool-down phase). The relative humidity in the experimental units was allowed to fluctuate during the heat period, but never to fall below 45%.

### 2.2. Dietary Treatments

Chickens were fed experimental diets throughout the trial with a starter diet for the first 10 days of housing, a grower diet from day 10 to day 24, and a finisher diet from day 25 to the end of the trial (day 43). The experimental diets were formulated according to two different nutritional specifications, the first meeting the minimal nutritional requirements for poultry by the National Research Council [13] (NRC) and the second meeting the Aviagen nutritional recommendations for Ross 308 broilers [14] (AVI). According to AVI recommendations, diets were additionally supplemented with: 200 IU vitamin E/kg (AVI + vitE), 250 mg vitamin C/kg (AVI + vitC), 0.20 mg Se/kg (AVI + Se), or a combination of 200 IU vitamin E, 250 mg vitamin C, and 0.20 mg Se/kg feed (AVI + ECSe). For supplementation, Rovimix E50 (DSM, Heerlen, The Netherlands) was used as vitamin E supplement (DL-α-tocopheryl acetate), Rovimix Stay-C35 (DSM, Heerlen, The Netherlands) as vitamin C supplement, and SelSaf 3000 (Lesaffre, Marcq en Baroeul, France) as Se supplement (organic Se, mainly L (+)-selenomethionine, produced by *Saccharomyces cerevisiae*). The components and calculated nutrient contents of the experimental starter, grower, and finisher diets are shown in Table 1.

Samples were taken from the finisher experimental diets to determine the proximate composition, concentration of Se, α-, γ + β-, and δ-tocopherol, MDA, antioxidant capacity of water-soluble (ACW) and lipid-soluble compounds (ACL), and fatty acid (FA) composition (Table 2).

### 2.3. Experimental Procedure, Sample Collection and Analyses

At 43 day of age, 12 birds per group were randomly selected, weighed, and sacrificed by stunning and bleeding. Organ and breast meat samples were collected from each chicken and the organs (heart, gizzard, pancreas, proventriculus, liver, and intestine) were weighed, while carcasses were weighed and then dissected into breast, legs, wings, back, and abdominal fat, according to the marketing standards for poultry meat described by Commission Regulation No. 543/2008. Measurements of pH and T were performed 15 min and 24 h post-mortem using a Mettler Toledo Seven2Go portable pH meter (Mettler Toledo, Schwerzenbach, Switzerland), while electrical conductivity was measured 24 h post-mortem using LF-Star (Ingenieurbüro Matthäus, Nobitz, Germany). Drip loss was measured 48 h post-mortem according to the method described in Voljč et al. [11] and calculated as the difference in weight between the 48 h and 24 h post-mortem meat samples enclosed in airtight bags and expressed as a percentage. Meat color was measured 24, 48, 72, 96, and 120 h post-mortem with a colorimeter CR 300 (Minolta Co., Ltd., Osaka, Japan) using the CIELAB system for lightness (L*), redness (a*), and yellowness (b*) as described by Voljč et al. [11]. The right part of the breast muscle was cut into 6 slices to measure the effects of different storage conditions, where 2 slices were untreated (fresh meat), 2 slices were stored in the refrigerator (at 4 °C for 6 days), and 2 slices were stored in the freezer (for 3 months at −20 °C). The chilled meat samples were packed in a tray with stretch film, and the frozen meat samples were packed in polypropylene plastic bags with a zipper. Prior to analysis, all samples were homogenized with a knife mill (Grindomix GM200, Retsch GmbH and Co., Haan, Germany) using liquid nitrogen to prevent oxidation.

Concentrations of α- and γ-tocopherol in feed and meat samples were determined according to the method described by Leskovec et al. [27], except that 300 mg of feed, 1.3 to 1.4 g of homogenized muscle, and 2 mL of hexane were used to extract tocopherols and no saponification was performed. Tocopherols extracted from the feed and breast muscle samples were analyzed by reverse-phase High Performance Liquid Chromatography (HPLC) (Agilent 1260 Infinity) using a Prodigy 5-μm ODS2 column (250 mm × 4.6 mm × 5 μm, Phenomenex, Torrance, CA, USA) with an Agilent 1260 Infinity FLD fluorescence detector.

The selenium content in fresh breast muscle samples was determined according to the method of the European standard EN 14.627, which specifies the determination of Se by hydride generation atomic absorption spectrometry according to the procedure described by Leskovec et al. [27].

The ACW and ACL in feed and ACW in breast muscle samples, respectively, were measured using PhotoChem (Analytik Jena, Jena, Germany) according to the ACW-Kit and ACL-Kit protocols provided by the manufacturer. The ACL procedure of the samples (0.2 g homogenized feed sample mixed with 1 mL hexane) is described in more detail by Rezar et al. [28].

Lipid oxidation of the breast muscle samples was determined by measuring the MDA levels according to the method described in Voljč et al. [11] and Rezar et al. [28], using HPLC. The determination of MDA in the homogenized feed and breast meat samples (0.1 g and 0.3 g, respectively) was performed using the external standard TEP and Agilent 1260 Infinity HPLC (Santa Clara, CA, USA) equipped with a 1260 Infinity FLD fluorescence detector.

The FA composition of the experimental diets and fresh breast muscle samples was determined by gas chromatography according to the method described by Voljč et al. [29]. An Agilent 6890 CG (Agilent, Santa Clara, CA, USA) with an Omegawax 320 column (30 m × 0.32 mm i.d. × 0.25 μm, Supelco, Bellefonte, PA, USA) and a flame ionization detector was used for the separation of FA methyl esters.

### 2.4. Statistical Analyses

Statistical analysis was performed using the MIXED procedure of SAS software Ver. 9.4 (SAS Institute Inc., Cary, NC, USA). Significant differences between least squares means (LSMEANS) of the experimental unit for each variable were determined using the Tukey–Kramer multiple comparison test. Dispersion was expressed as the standard error of the mean (SEM). In the statistical model, experimental groups were set as a fixed effect, and the replication pens were set as a random effect. For continuous measurements, storage time was included in the model as a fixed effect. Statistical significance was set at *p* < 0.05.

## 3. Results and Discussion

The health status and mortality of broilers were monitored daily throughout the trial. During the experimental period, the birds adapted well to the experimental conditions and had no health problems. Due to the number of birds in the trial, performance parameters should only be considered indicative of the trial conditions and, as expected, are somewhat lower than the Aviagen growth objectives due to HS. Previously, it was reported that the exposure of broilers to HS conditions impairs their growth performance, which was manifested as a reduction in FI, feed efficiency, BWG and final body weight (BW) [4]. In the present study, the dietary antioxidants alone or in combination had no major effect on the performance characteristics in terms of BWG, FCR, and FI (data not shown). On the other hand, the BW of broilers fed according to the NRC [13] requirements were significantly lower (*p* < 0.05) than the BW of broilers fed according to the Aviagen [14] recommendations (average of all five AVI groups) on day 28 (1255 g compared to 1402 g), day 35 (1732 g compared to 1965 g), and at the end of the experiment (2174 g compared to 2487 g).

### 3.1. Carcass Traits

Carcass characteristics, together with meat quality traits, are important in poultry production, which strives to avoid product defects and thus provide a product suitable for consumer acceptance [30]. In the present study, the carcass yield and yields of breast, legs, wings and back were not affected by the dietary supplementations (Table 3). Carcass, breast, and tight yields were similar in all experimental groups and did not deviate from the performance objectives established for Ross 308 broilers. This is consistent with other studies in which the dietary supplementation with vitamins E and C [31] and vitamin E and Se [5] had no effect on the carcass, breast, and thigh yields. Similarly, Leskovec et al. [25] reported that the dietary supplementation with vitamins E, C, and Se, or their combination, did not cause differences in the carcass dressing and traits in broilers under oxidative stress induced by a high PUFA intake, although the breast yield was higher in the group of broilers receiving 200 IU vitamin E/kg of feed. This agrees with the results of Wu et al. [32], who showed a significant increase in the breast yield by vitamin E supplementation (60 or 120 mg/kg feed), whereas the eviscerated carcass yield was not affected.

In our study, the abdominal fat was not affected by the cyclic HS or the dietary treatments, which is not consistent with the study by Habiban et al. [5], who reported that different levels of vitamin E linearly decreased the abdominal fat yield of broilers under HS. On the other hand, our results are in agreement with those of Zeferino et al. [31], in which the abdominal fat yield of broilers under the conditions of HS was not affected by the supplementary combination of vitamin E and vitamin C, Peña et al. [33], who found no effect of different levels of dietary ascorbic acid and citric flavonoids on the abdominal fat yield of HS broilers, and Habiban et al. [5], where different levels of Se supplementation had no effect on the abdominal fat yield. In contrast to our results, the dietary inclusion of vitamins and microelements reduced the abdominal fat content of broilers exposed to HS in a study by Kucuk et al. [34]. Furthermore, it has been reported that the exposure of poultry to high ambient temperatures caused a decrease in the fat and protein deposition in the breast muscle due to increased muscle protein breakdown, decreased muscle protein synthesis [31], and a higher percentage of abdominal fat. Abdominal fat is an important parameter indicating an excessive fat deposition in broilers, which negatively affects the carcass appearance and is associated with the reduction in the basal metabolic rate and lower physical activity of birds [35]. In addition, HS causes an increase in the plasma corticosterone concentration, which is reported to increase the fat accumulation in the abdominal, cervical, and thigh adipose tissues [36].

In the present study, the different dietary supplements had no effect on the absolute and relative weights of the heart, liver, pancreas, proventriculus, and intestine (data not shown). This is consistent with the study by Zeferino et al. [31], which reported that the dietary supplementation with a combination of vitamins E and C had no effect on the organ characteristics in heat-stressed broilers, and with the study by Habibian et al. [15], in which different levels of dietary Se did not improve the liver and lymphoid organ weights, and in which no interaction between vitamin E, Se, or ambient temperature was observed for the relative organ weights.

### 3.2. Meat Quality Parameters

One of the most commonly accepted factors for predicting the technological and sensory qualities of meat is the pH. Heat exposure prior to slaughter can lead to a rapid decrease in the muscle pH due to an accelerated postmortem glycolysis, whereas the muscle temperature is still high, resulting in the appearance of PSE meat [2]. This is associated with lipid peroxidation, increased cooking and drip loss, and reduced WHC [37]. In the current study, no effects of the dietary supplementation were observed on the pH and temperature measured 15 min and 24 h post mortem, drip loss, and electrical conductivity of the breast muscle (Table 4). Similarly to our results, Mazur-Kuśnirek et al. [10] reported that different levels of vitamin E had no effect on breast muscle pH values measured 15 min and 24 h post mortem, although decreased drip loss and improved WHC were measured in breast meat of broilers under HS receiving vitamin E, compared to non-supplemented ones. On the other hand, Zdanowska-Sasiadek et al. [38] observed that the addition of dietary vitamin E resulted in higher pH_24h_, lower cooking loss, and better sensory quality of fresh and stored breast meat. In addition, Ferreira et al. [39] reported that vitamin C had no effect on the pH of broiler thigh meat. It was also reported that the supplementation with vitamin C had a positive effect on the breast meat pH [40]. In addition, Wang et al. [41] observed no effects of different dietary Se sources on the breast muscle pH, shear force, and drip loss, which is consistent with our results.

The stability of breast meat color is influenced by many different factors, such as genotype, gender, and slaughter age of the birds, environmental conditions, pre-slaughter stress, muscle pH, myoglobin concentrations, etc. [42]. Contrary to our assumptions, the breast meat color in the present trial was not affected by the different dietary treatments (Table 5). Those findings are consistent with the study by Hashizawa et al. [43], where neither HS nor dietary vitamin E affected the meat color, although the storage time decreased the redness (a*) and increased the breast muscle brightness (L*) and yellowness (b*). This partially agrees with our results, where the breast meat redness was significantly decreased by the storage at 4 °C in the NRC, AVI, and AVI + vitE groups, compared to the other experimental groups. In contrast, the dietary supplementation of vitamin E above recommended levels has been reported to improve the meat quality by reducing the lipid oxidation in skeletal muscle, which positively affects the discoloration of the meat by delaying the oxidation of myoglobin or oxymyoglobin to metmyoglobin [44]. Similar to our results, Zhang et al. [45] reported that the dietary addition of vitamin E did not cause differences in the broiler meat color, which the authors attributed to nonsignificant changes in the breast muscle pH. As in our study, Skřivan et al. [46] reported no effect of the dietary vitamin C supplementation on the color and other sensory characteristics of breast and tight chicken meat, whereas Niu et al. [18] reported a positive effect of dietary vitamin C on the yellowness of breast muscle, WHC and share force of the tight muscle and consequently the tenderness of the meat. Furthermore, contrary to the present study, Khan et al. [47] reported that the dietary Se supplementation increased the a* and b* values and decreased the L* values, as well as improved the sensory properties of chicken breast meat. Consistent with the results of our previous study [25], we did not detect a synergistic effect of the dietary antioxidants on the breast meat quality parameters, suggesting that the supranutritional addition of vitamins E, C, and Se, supplemented alone or in combination, did not affect post-mortem processes in the breast meat.

### 3.3. Oxidative Stability of Breast Meat

Under stress conditions, especially HS, α-tocopherol radicals are rapidly oxidized, resulting in lower levels of tocopherols in the meat. In our study, the supplementation with α-tocopheryl acetate significantly elevated the content of α-tocopherol in the breast meat samples in both groups, AVI + vitE and AVI + ECSe (Table 6).

The dietary supplementation with Se is reported to increase the Se content in meat, while organic Se sources have a better absorption efficiency than inorganic Se sources due to different metabolic pathways, suggesting that organic Se is more efficiently deposited in tissues [48]. In the present study, the addition of organic dietary Se to broiler diets significantly increased the Se content in fresh breast meat, and the addition of all three antioxidants was even more effective in accumulating Se in muscle tissues, indicating a possible sparing effect of vitamins E and C on Se-dependent enzymes, and consequently higher Se content in muscle tissues. Similarly, a study by Habibian et al. [5] showed that the Se content in breast meat was increased by the vitamin E supplementation in broilers under HS.

In agreement with our previous results [25,27], we did not observe differences in the content of ACW, predominantly measuring vitamin C, in the breast meat, which could be attributed to the rapid metabolism and secretion of vitamin C and the possible exchange with other water-soluble antioxidants. The duration and different temperatures of storage did not affect the ACW content in breast meat (data not shown).

Chicken breast meat contains low levels of saturated fatty acids (SFA) and high levels of unsaturated FAs, especially PUFA, and is therefore more susceptible to lipid oxidation, leading to a higher risk of oxidative rancidity in the muscle tissue of live birds and persisting in the fresh carcass and in stored and processed meat products [44]. The extent of the lipid oxidation in poultry meat is often influenced by several factors, such as the lipid profile of the meat, storage temperature, and heat treatment [29]. In our study, the lowest MDA content, as a secondary by-product of the lipid peroxidation, was found in the chilled breast meat of the vitamin E-supplemented groups: 92.0% lower in the AVI + vitE group and 86.1% lower in the AVI + ECSe group than in the NRC group, and also 90.8% lower in the AVI + vitE group than in the AVI + Se group. However, no differences were found in the fresh meat between the experimental groups and between the different storage processes. This is comparable to our previous studies [11,25], in which the addition of 200 IU vitamin E/kg resulted in a significantly lower MDA content in raw, stored, and cooked chicken breast meat. Moreover, the addition of vitamin C and Se had no significant effect on the MDA content in breast meat, which is in agreement with the results of Leskovec et al. [25], where concentrations equal to the present study (250 mg/kg vitamin C and 0.2 mg Se/kg) of dietary ascorbic acid and Se had no effect on the MDA content in the breast muscle. Although previous studies reported that combinations of vitamin E and vitamin C [49], and vitamin E and Se [5], act synergistically to prevent lipid peroxidation under HS, no synergistic effect of the dietary antioxidants was observed in the present study. Although there is no limit to MDA concentration in meat, several studies have confirmed that MDA levels below 0.50 mg/kg are acceptable in terms of oxidation processes [50]. In the present study, the MDA concentration in the breast meat samples did not reach the above-mentioned value, so we can assume that the overall oxidative stability of the breast meat was not affected, regardless of the storage process and dietary supplementation.

### 3.4. Fatty Acid Composition of Breast Muscle

In the present study, the FA composition of the breast muscle was not affected by the different dietary supplements (Table 7). This complies with the study by Zdunczyk et al. [51], who observed no differences in the FA composition of the breast muscle when different combinations of supranutritive levels of vitamin E (from 40 to 200 mg/kg) and Se (0.15 and 0.50, respectively) were added to broiler diets. In contrast, Mazur-Kuśnirek et al. [10] found that the supplementation with higher levels of vitamin E prevented the oxidation of PUFA and decreased the content of MUFA in the breast muscles of broilers exposed to high temperatures. Furthermore, Pappas et al. [24] found that the supplementation with higher dietary levels of organic Se (0.15, 0.3, and 3 mg/kg) in chicken breast meat resulted in the preservation of the long-chain PUFA in a linear manner. The FA profile of chicken meat is variable and influenced by the amount and composition of lipids and FA present in their diet. In turn, the effects of different dietary antioxidants on the FA content of chicken meat are rather inconsistent, so further trials on this area of investigation are recommended.

## 4. Conclusions

The results of the present study suggest that both the Aviagen recommendations for Ross 308 broilers and the NRC requirements are comparable in mitigating the negative effects of HS on meat quality and oxidative stability of breast meat. The addition of supranutritional levels of α-tocopherol and selenium above the Aviagen recommendations significantly increased their content in breast muscle, while the supplementation with α-tocopherol showed a protective role in preventing oxidative damage to lipids and improving meat stability during the storage process. In general, the Aviagen recommendations for antioxidants are adequate for broilers under HS, with the exception of the supranutritional supplementation with vitamin E, which further reduces lipid oxidation in breast meat. No obvious synergistic effects of the added antioxidants were observed. Furthermore, the addition of supranutrtional levels of the antioxidants studied had no adverse effects when supplemented either alone or in combination. In conclusion, further studies would be of great interest to define the mechanisms of the potential synergistic effects of vitamins E, C, and Se in relation to other environmental stressors and dietary antioxidant levels.

## Figures and Tables

**Table 1:** Composition and calculated nutrient content of the experimental diets for broiler chickens.

Component	Starter (d 1–10)	Grower (d 11–24)	Finisher (d 25–43)
**Composition of diets ^1^**			
Maize (g/kg)	314	360	515
Wheat (g/kg)	200	200	100
Wheat bran (g/kg)	30.0	15.0	0.00
Soya meal (g/kg)	274	234	193
Corn gluten meal (g/kg)	85.0	91.2	93.0
Plant oil (g/kg)	50.8	56.1	57.2
Salt (g/kg)	4.58	4.95	5.00
Monocalcium phosphate (g/kg)	12.4	8.20	9.00
Limestone (g/kg)	15.6	17.5	15.3
L-lysine-HCl (g/kg)	5.25	4.68	4.61
DL-methionine (g/kg)	1.58	2.20	1.95
L-threonine (g/kg)	1.45	1.15	0.90
Mineral-vitamin supplement ^2^ (g/kg)	5.00	5.00	5.00
**Calculated energy and nutrient content**			
Metabolizable energy (MJ/kg)	12.6	13.0	13.4
Crude protein (g/kg)	228	214	194
Lysine (g/kg)	12.9	11.5	10.3
Methionine (g/kg)	5.12	5.61	5.19
Calcium (g/kg)	8.95	8.70	7.89
Phosphorus-available (g/kg)	5.45	4.36	4.38

^1^ Feed mixtures also contain coccidiostat, Maxiban^®^ G160, Elanco Products Co., Hook, Hampshire, UK. ^2^ Calculated to meet the mineral and vitamin requirement for NRC finisher diets or Ross 308 finisher diet.

**Table 2:** Proximate composition, concentration of Se, α-, γ + β-, and δ-tocopherol, MDA, and antioxidant capacity of lipid- (ACL) and water- (ACW) soluble compounds and the fatty acid (FA) composition of the finisher experimental diets.

Component	Diets					
	NRC	AVI	AVI + vitE	AVI + vitC	AVI + Se	AVI + ECSe
Dry matter (g/kg)	893	893	893	894	894	896
Crude protein (g/kg)	198	199	194	190	195	194
Crude fat (g/kg)	80.1	78.6	78.8	80.2	78.4	81.7
Crude ash (g/kg)	49.8	49.1	47.0	48.6	49.6	51.2
Crude fiber (g/kg)	28.2	27.7	28.1	30.2	26.2	25.9
Se (mg/kg)	0.14	0.25	0.21	0.23	0.46	0.48
**Tocopherol isomers**						
α-tocopherol (mg/kg)	18.6	62.7	226	62.8	60.7	242
γ + β-tocopherol (mg/kg)	25.6	25.6	26.1	23.3	23.2	25.8
δ-tocopherol (mg/kg)	1.35	1.42	1.35	1.39	1.42	1.22
**ACL, ACW, MDA**						
ACL (µmol/kg)	0.50	0.48	0.50	0.49	0.50	0.60
ACW (µmol/kg)	1.38	0.87	0.79	0.86	0.68	0.79
MDA (nmol/g)	1.30	1.40	1.50	1.60	1.70	1.50
**Fatty acid composition ^1^ (g of FA/100 g FAs)**						
C16:0	21.0	21.1	20.9	21.2	21.1	20.2
∑ C16:1 ^2^	0.25	0.26	0.25	0.25	0.26	0.26
C18:0	37.7	37.8	37.7	38.1	37.8	38.4
∑ C18:1 ^2^	33.3	33.2	33.7	32.9	33.3	33.7
C18:2 n-6	2.36	2.28	2.25	2.27	2.27	2.29
C18:3 n-3	0.42	0.42	0.42	0.42	0.41	0.40
Sum of SFA ^3^	25.9	25.9	25.7	26.1	25.9	24.9
Sum of MUFA ^4^	38.4	38.5	38.4	38.8	38.5	39.08
Sum of PUFA ^5^	35.7	35.5	35.9	35.2	35.6	36.0
n-3 PUFA ^5^	2.36	2.28	2.25	2.27	2.27	2.29
n-6 PUFA ^5^	33.3	33.3	33.7	32.9	33.3	33.7
n-6/n-3 PUFA ^5^	14.2	14.6	14.9	14.5	14.7	14.7

NRC = recommended levels of NRC, no supplementation, AVI = recommended levels of Aviagen, no supplementation, AVI + vitE = AVI + 200 IU DL-α-tocopheryl acetate/kg feed, AVI + vitC = AVI + 250 mg vitamin C/kg feed, AVI + Se = AVI + 0.2 mg Se/kg feed, AVI + ECSe = AVI + vitE + vitC + Se. ^1^ Values represent means of 2 analyses per sample. Only the prevalent and dietary important fatty acids are listed. ^2^ Sum of isomers. ^3^ Saturated fatty acid. ^4^ Monounsaturated fatty acid. ^5^ Polyunsaturated fatty acid.

**Table 3:** Effect of the dietary supplements on the dressing percentage and carcass yields (%) of breast and leg muscles, wings, back and abdominal fat at the end of the experiment.

	Diets						SEM	*p*-Value
	NRC	AVI	AVI + vitE	AVI + vitC	AVI + Se	AVI + ECSe		
	Percentage of BW							
Carcass yield (%)	76.2	76.1	76.5	76.7	76.9	76.8	0.49	0.809
Breast muscle (%)	36.2	36.1	36.1	36.6	36.7	36.2	0.71	0.968
Leg muscles (%)	30.4	30.1	29.7	29.9	29.8	30.2	0.46	0.886
Wings (%)	11.3	11.1	11.0	10.7	11.0	10.6	0.16	0.133
Back (%)	20.3	20.6	20.5	21.0	19.9	21.3	0.55	0.568
Abdominal fat (%)	1.90	2.12	1.86	2.05	1.94	1.72	0.14	0.417

Nomenclature of the experimental groups as in Table 2.

**Table 4:** Effect of the diet supplementation with vitamins E and C and Se on the pH values, temperature, drip loss, and electrical conductivity of the breast muscle.

	Diets						SEM	*p*-Value
	NRC	AVI	AVI + vitE	AVI + vitC	AVI + Se	AVI + ECSe		
pH_15min_	6.32	6.47	6.39	6.29	6.40	6.41	0.05	0.190
pH_24h_	6.06	6.13	6.19	6.19	6.20	6.18	0.04	0.187
T (°C)_15min_	40.9	41.4	41.4	41.2	41.1	41.2	0.19	0.561
T (°C)_24h_	5.88	5.43	5.08	5.55	5.34	5.65	0.27	0.507
Drip loss (%)	0.51	0.58	0.64	0.57	0.57	0.56	0.04	0.410
Elect. Cond. ^1^ (S/m)	4.95	3.90	5.01	3.87	3.98	4.44	0.34	0.182

Nomenclature of the experimental groups as in Table 2. ^1^ Electrical conductivity.

**Table 5:** Effect of the diet supplementation with vitamins E and C, and Se on the breast meat color of broiler chickens during 5 days of refrigerated storage (4 ± 1 °C).

	Hours	Diets						SEM	*p*-Value
		NRC	AVI	AVI + vitE	AVI + vitC	AVI + Se	AVI + ECSe		
L* ^1^	24 h	48.7	49.6	48.7	48.7	47.3	48.7	0.84	0.623
	48 h	48.2	49.6	49.5	49.2	48.9	49.1	0.84	0.853
	72 h	48.3	48.8	49.8	49.2	48.5	47.8	0.80	0.620
	96 h	48.0	50.1	48.8	48.9	47.5	49.2	0.71	0.293
	120 h	47.6	48.4	48.6	48.7	48.8	47.9	0.70	0.791
*p*-Value (time)		0.814	0.418	0.808	0.958	0.633	0.438		
a* ^2^	24 h	3.94 ^A^	2.64 ^A^	3.83 ^A^	2.91	3.53	2.90	0.26	0.087
	48 h	3.58 ^AB^	2.08 ^AB^	2.63 ^B^	2.63	2.60	2.60	0.28	0.111
	72 h	2.91 ^AB^	1.99 ^AB^	2.35 ^B^	2.24	2.81	2.14	0.28	0.266
	96 h	2.91 ^AB^	1.60 ^B^	2.49 ^B^	2.03	2.32	2.18	0.26	0.129
	120 h	2.82 ^B^	1.68 ^B^	2.55 ^B^	1.85	2.68	2.33	0.26	0.104
*p*-Value (time)		0.013	0.010	0.001	0.069	0.107	0.242		
b* ^3^	24 h	13.6	12.4	12.1	11.4	11.9	12.16	0.42	0.492
	48 h	14.5	12.7	12.3	12.3	12.0	12.29	0.48	0.062
	72 h	13.7	13.1	12.2	12.0	11.8	11.95	0.41	0.079
	96 h	14.4	13.4	12.7	12.4	12.7	12.73	0.49	0.159
	120 h	13.8	12.9	12.8	12.0	12.6	12.15	0.55	0.335
*p*-Value (time)		0.546	0.338	0.521	0.570	0.747	0.681		

Nomenclature of the experimental groups as in Table 2. ^1^ L*–Lightness, ^2^ a*–Redness, ^3^ b*–Yellowness, measured on consecutive days post-mortem: 24 h, 48 h, 72 h, 96 h, and 120 h. ^A,B^ Different superscript letters within the column and parameter show significant differences (*p* < 0.05).

**Table 6:** Content of α- and γ-tocopherol, MDA, ACW, and Se in the fresh, chilled, and frozen stored breast meat.

	Diets						SEM	*p*-Value
	NRC	AVI	AVI + vitE	AVI + vitC	AVI + Se	AVI + ECSe		
**Fresh breast meat**								
α-tocopherol (µg/100 g)	265.1 ^a^	490.2 ^a^	1432.3 ^b^	585.4 ^a^	515.4 ^a^	1515.9 ^b^	85.23	0.0002
γ-tocopherol (µg/100 g)	58.8	53.0	48.4	60.9	52.3	49.3	6.56	0.739
Se (µg/100 g)	988.7 ^a^	923.3 ^a^	931.8 ^a^	870.6 ^a^	1980.7 ^b^	2119.8 ^c^	38.50	<.0001
ACW ^1^ (µmol AA ^2^/100 g)	19.3	23.1	25.1	23.4	21.8	21.4	2.29	0.612
MDA ^3^ (mg/kg)	0.179	0.055	0.032	0.056	0.063	0.016	0.05	0.3498
**Chilled ^4^ breast meat**								
α-tocopherol (µg/100 g)	277.4 ^a^	460.3 ^b^	1427.5 ^c^	536.7 ^b^	520.1 ^b^	1596.9 ^d^	32.19	<.0001
γ-tocopherol (µg/100 g)	61.1	55.9	53.8	57.1	54.4	55.8	3.86	0.838
ACW ^1^ (µmol AA ^2^/100 g)	10.3	26.3	26.1	20.2	18.6	21.5	3.10	0.107
MDA ^3^ (mg/kg)	0.062 ^a^	0.036 ^abc^	0.006 ^b^	0.024 ^abc^	0.054 ^ac^	0.009 ^bc^	0.01	0.017
**Frozen stored ^5^ breast meat**								
α-tocopherol (µg/100 g)	291.9 ^a^	543.6 ^b^	1568.0 ^c^	542.8 ^b^	596.2 ^b^	1746.0 ^c^	41.38	<.0001
γ-tocopherol (µg/100 g)	63.9	61.2	58.0	57.3	63.5	59.8	4.23	0.719
ACW ^1^ (µmol AA ^2^/100 g)	16.9	24.2	23.0	24.8	21.6	23.9	1.35	0.081
MDA ^3^ (mg/kg)	0.110 ^a^	0.030 ^b^	0.015 ^b^	0.032 ^b^	0.029 ^b^	0.019 ^b^	0.01	0.013

Nomenclature of the experimental groups as in Table 2. ^1^ Antioxidant capacity of water-soluble antioxidants. ^2^ Ascorbic acid. ^3^ Malondialdehyde. ^4^ Breast meat stored in refrigerator at 4 °C for 6 d. ^5^ Breast meat stored in freezer at −20 °C for 3 months. ^a–d^ Different superscript letters within the row show significant differences (*p* < 0.05).

**Table 7:** Fatty acid profile of the fresh breast muscle (g FA/100 g total FAs).

	Diets						SEM	*p*-Value
	NRC	AVI	AVI + vitE	AVI + vitC	AVI + Se	AVI + ECSe		
**Fatty acids ^1^ (g of FA/100 g FAs)**								
C16:0	21.54	22.53	22.26	23.11	22.54	22.34	0.38	0.202
∑ C16:1 ^2^	2.06	3.73	3.05	3.59	3.06	3.46	0.26	0.059
C18:0	6.83	5.94	6.25	5.84	6.30	5.43	0.40	0.321
∑ C18:1 ^2^	31.28	33.28	34.11	34.78	35.03	35.47	0.97	0.100
C18:2 n-6	23.05	21.83	21.30	21.95	22.10	22.89	0.61	0.368
C18:3 n-3	1.19	1.25	1.14	1.26	1.31	1.40	0.06	0.182
C20:4 n-6	5.35	3.79	4.23	3.23	3.24	2.77	0.52	0.075
C20:5 n-3	0.15	0.13	0.16	0.10	0.11	0.08	0.02	0.164
C22:4 n-6	1.11	0.86	1.12	0.76	0.79	0.64	0.11	0.092
C22:5 n-3	0.88	0.61	0.72	0.55	0.52	0.50	0.10	0.144
C22:6 n-3	0.62	0.44	0.58	0.38	0.40	0.37	0.07	0.104
Sum of SFA ^3^	31.06	31.44	31.38	31.60	30.56	30.67	0.40	0.516
Sum of MUFA ^4^	37.06	38.16	36.73	38.85	38.59	39.86	1.56	0.733
Sum of PUFA ^5^	31.13	29.25	29.51	29.27	28.98	29.25	1.32	0.848
n-3 PUFA ^5^	29.22	27.48	27.75	27.71	27.23	27.32	1.25	0.855
n-6 PUFA ^5^	2.62	2.49	2.71	2.35	2.39	2.40	0.21	0.797
n-6/n-3 PUFA ^5^	11.30	11.23	10.59	11.52	11.55	11.23	0.52	0.788

Nomenclature as in Table 2. ^1^ Values represent the means of 2 analyses per sample. Only the prevalent and dietary important fatty acids are listed, whereas the sum of SFA, MUFA, and PUFA are calculated from all analyzed fatty acids. ^2^ Sum of isomers. ^3^ Saturated fatty acids. ^4^ Monounsaturated fatty acids. ^5^ Polyunsaturated fatty acids.

## Data Availability

Not applicable.
